# Non-maintenance intravesical Bacillus Calmette–Guérin induction therapy with eight doses in patients with high- or highest-risk non-muscle invasive bladder cancer: a retrospective non-randomized comparative study

**DOI:** 10.1186/s12885-021-07966-7

**Published:** 2021-03-11

**Authors:** Makito Miyake, Kota Iida, Nobutaka Nishimura, Tatsuki Miyamoto, Kiyohide Fujimoto, Ryotaro Tomida, Kazumasa Matsumoto, Kazuyuki Numakura, Junichi Inokuchi, Shuichi Morizane, Takahiro Yoneyama, Yoshiaki Matsumura, Takashige Abe, Masaharu Inoue, Takeshi Yamada, Naoki Terada, Shuya Hirao, Motohide Uemura, Yuto Matsushita, Rikiya Taoka, Takashi Kobayashi, Takahiro Kojima, Yoshiyuki Matsui, Hiroshi Kitamura, Hiroyuki Nishiyama, on behalf of the Japanese Urological Oncology Group

**Affiliations:** 1 Department of Urology, Nara Medical University, 840 Shijo-cho, Nara, 634-8522 Japan; 2 Department of Urology, National Hospital Organization Shikoku Cancer Center, Matsuyama, Ehime Japan; 3 Department of Urology, Kitasato University School of Medicine, Sagamihara, Kanagawa Japan; 4 Department of Urology, Akita University Graduate School of Medicine, Akita, Japan; 5 Department of Urology, Graduate School of Medical Sciences, Kyushu University, Fukuoka, Japan; 6 Division of Urology, Department of Surgery, Faculty of Medicine, Tottori University, Tottori, Japan; 7 Department of Advanced Transplant and Regenerative Medicine, Hirosaki University Graduate School of Medicine, Hirosaki, Aomori Japan; 8 Department of Urology, Nara Prefecture General Medical Center, Nara, Japan; 9 Department of Urology, Hokkaido University Graduate School of Medicine, Sapporo, Hokkaido Japan; 10 Department of Urology, National Cancer Center Hospital East, Chiba, Japan; 11 Department of Urology, Kyoto Prefectural University of Medicine, Kyoto, Japan; 12 Department of Urology, Miyazaki University, Miyazaki, Japan; 13 Department of Urology, Hirao Hospital, Kashihara, Nara, Japan; 14 Department of Urology, Osaka University, Graduate School of Medicine, Suita, Osaka, Japan; 15 Department of Urology, Hamamatsu University School of Medicine, Shizuoka, Japan; 16 Department of Urology, Faculty of Medicine, Kagawa University, Takamatsu, Kagawa Japan; 17 Department of Urology, Kyoto University Graduate School of Medicine, Kyoto, Japan; 18 Department of Urology, Faculty of Medicine, University of Tsukuba, Tsukuba, Ibaraki, Japan; 19 Department of Urology, National Cancer Center Hospital, Tokyo, Japan; 20 Department of Urology, Faculty of Medicine, University of Toyama, Toyama, Japan

**Keywords:** Urinary bladder neoplasms, Non-muscle invasive bladder cancer, Bacillus Calmette-Guérin (BCG), Intravesical therapy, Propensity score matching, Landmark analysis

## Abstract

**Background:**

To explore possible solutions to overcome chronic Bacillus Calmette–Guérin (BCG) shortage affecting seriously the management of non-muscle invasive bladder cancer (NMIBC) in Europe and throughout the world, we investigated whether non-maintenance eight-dose induction BCG (iBCG) was comparable to six-dose iBCG plus maintenance BCG (mBCG).

**Methods:**

This observational study evaluated 2669 patients with high- or highest-risk NMIBC who treated with iBCG with or without mBCG during 2000–2019. The patients were classified into five groups according to treatment pattern: 874 (33%) received non-maintenance six-dose iBCG (Group A), 405 (15%) received six-dose iBCG plus mBCG (Group B), 1189 (44%) received non-maintenance seven−/eight-dose iBCG (Group C), 60 (2.2%) received seven−/eight-dose iBCG plus mBCG, and 141 (5.3%) received only ≤5-dose iBCG. Recurrence-free survival (RFS), progression-free survival, and cancer-specific survival were estimated and compared using Kaplan–Meier analysis and the log-rank test, respectively. Propensity score-based one-to-one matching was performed using a multivariable logistic regression model based on covariates to obtain balanced groups. To eliminate possible immortal bias, 6-, 12-, 18-, and 24-month conditional landmark analyses of RFS were performed.

**Results:**

RFS comparison confirmed that mBCG yielded significant benefit following six-dose iBCG (Group B) in recurrence risk reduction compared to iBCG alone (groups A and C) before (*P* < 0.001 and *P* = 0.0016, respectively) and after propensity score matching (*P* = 0.001 and *P* = 0.0074, respectively). Propensity score-matched sequential landmark analyses revealed no significant differences between groups B and C at 12, 18, and 24 months, whereas landmark analyses at 6 and 12 months showed a benefit of mBCG following six-dose iBCG compared to non-maintenance six-dose iBCG (*P* = 0.0055 and *P* = 0.032, respectively). There were no significant differences in the risks of progression and cancer-specific death in all comparisons of the matched cohorts.

**Conclusions:**

Although non-maintenance eight-dose iBCG was inferior to six-dose iBCG plus mBCG, the former might be an alternative remedy in the BCG shortage era. To overcome this challenge, further investigation is warranted to confirm the real clinical value of non-maintenance eight-dose iBCG.

**Supplementary Information:**

The online version contains supplementary material available at 10.1186/s12885-021-07966-7.

## Background

Non-muscle-invasive bladder cancer (NMIBC) is a heterogeneous disease accounting for approximately 70% of initially diagnosed bladder cancers [1]. The recently updated European Association of Urology (EAU) guidelines stratify NMIBC patients into low-, intermediate-, high-, and highest-risk groups [2]. Immediate radical cystectomy should be considered for the highest-risk group; however, clinical practice guidelines suggest that intravesical instillation of Bacillus Calmette–Guérin (BCG) is a standard therapeutic and adjuvant option after transurethral resection of bladder tumor (TURBT) for high−/highest-risk disease [2–4]. A recent systematic review concluded that maintenance BCG (mBCG) following induction BCG (iBCG) was superior to non-maintenance iBCG, with reduced risks of tumor recurrence and progression [5]. However, other systematic reviews demonstrated that mBCG was not superior to non-maintenance iBCG regarding clinical outcomes despite an increased risk of BCG-induced toxicity [6, 7].

In Europe, the United States, and throughout the world, “chronic BCG shortage” is an emerging problem with BCG use [8, 9]. A significant shortage of the Tice strain (Merck, USA) with declining supplies over time was announced in 2014 and production of the Connaught strain (Sanofi Pasteur, Canada) was permanently closed in 2017 [9]. Weekly iBCG for 6 weeks followed by weekly mBCG for 3 weeks at 3, 6, 12, 18, 24, 30, and 36 months required no less than 27 doses for completion [10]. The scheduled duration of mBCG and the number of BCG doses (range, 12–27) vary among physicians and trials [5]. To face the era of BCG shortage, the optimal dosing schedule of iBCG and mBCG should be reconsidered.

While the six-dose iBCG is common in Europe and the United States, the eight-dose iBCG is frequently and traditionally used in Japan [11–13]. There are sparse data comparing oncological outcomes between six-dose iBCG with/without mBCG and non-maintenance eight-dose iBCG. This study investigated whether non-maintenance eight-dose iBCG was comparable to six-dose iBCG with mBCG.

## Methods

### Data collection

This retrospective multicenter study was approved by the institutional review board of each participating institute (reference protocol ID: 2217) of the Japan Urological Oncology Group framework. Informed consent was obtained from participants through posters and/or website using the opt-out method (https://www.ncbi.nlm.nih.gov/pmc/articles/PMC3079702/). We reviewed 3226 patients who received intravesical BCG for pathologically diagnosed NMIBC and treatment during 2000–2019 at 31 collaborative hospitals. The clinicopathological characteristics of the patients included age, sex, performance status, past history of NMIBC, tumor multiplicity, tumor size, T category, tumor grade (per 2004 World Health Organization classification), second TUR, presence of bladder carcinoma in situ (CIS) and prostatic urethra-involving CIS, divergent differentiation, e.g. squamous differentiation and glandular differentiation, variant histology, e.g. nested, micropapillary, and plasmacytoid variants [14], lymphovascular involvement (LVI). Depending on the clinicopathological characteristics, patients were stratified into the low-, intermediate-, high-, and highest-risk groups according to the updated EAU guidelines [2].

### Intravesical BCG treatment after TURBT

The criteria, dose, and schedule for iBCG and mBCG were not consistent and implemented at the physician’s discretion. Generally, most patients with high−/highest-risk NMIBC, such as papillary Ta/T1 high-grade tumors and CIS, were treated with intravesical BCG after TURBT. The intravesical BCG schedule included weekly instillations of Immunobladder (80 mg of Tokyo-172 strain) or ImmuCyst (81 mg of Connaught strain, currently unavailable) for 6–8 consecutive weeks with or without subsequent mBCG. mBCG was administered once a week for 3 weeks at 3, 6, 12, 18, 24, 30, and 36 months after iBCG initiation [10]. In this study, at least 2 doses out of 3 doses in the first mBCG round at 3 months was considered mBCG implementation.

### Stratification of the study cohort based on intravesical BCG treatment

**Fig. 1:**
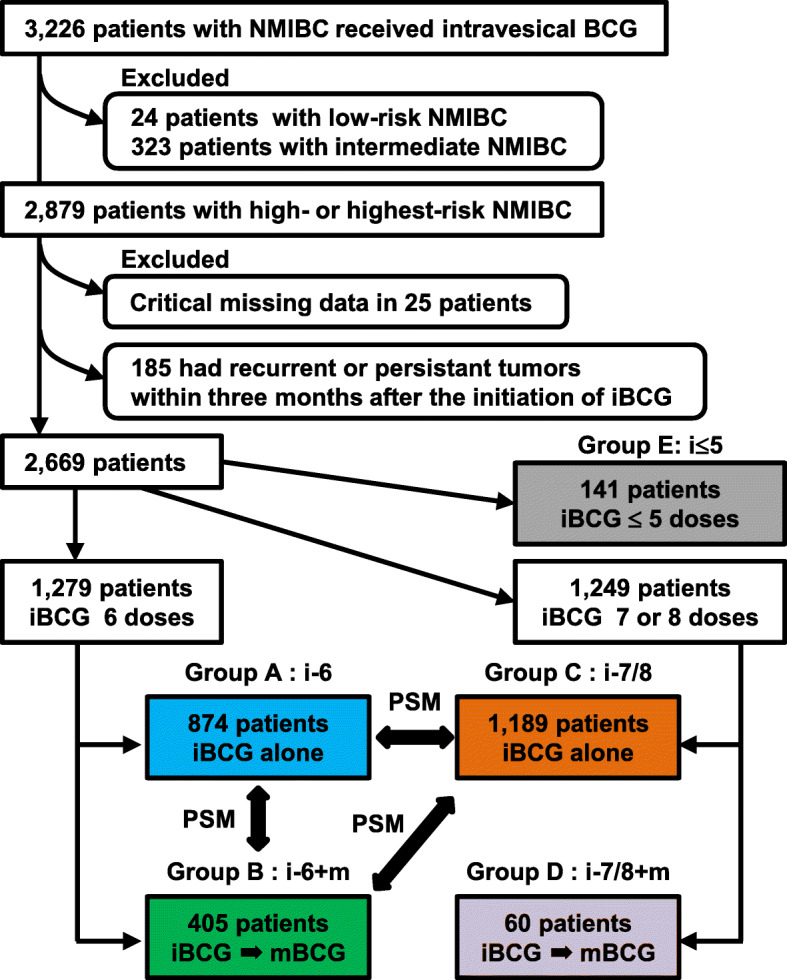
Flow chart for creation of the patient cohort dataset. Patients were stratified according to the 2019 European Association of Urology NMIBC guidelines into four as follows: low-, intermediate-, high-, and highest-risk groups. Abbreviations: NMIBC, non-muscle invasive bladder cancer; BCG, Bacillus Calmette–Guérin; iBCG, induction BCG; mBCG, maintenance BCG; PSM, propensity score matching

Figure 1 shows a flowchart of patient selection process. Of 3226 patients, the cohort was first restricted to 2879 patients (89%), including only patients with high−/highest-risk NMIBC. Next, 25 patients (0.8%) with critical missing data and 185 patients (6.4%) with recurrent or persistent tumors within 3 months after iBCG initiation were excluded. Among 185 patients, 9 (5%) received < 5 doses, 89 (48%) received 6 doses, and 82 (44%) received 7 or 8 doses of iBCG. The remaining 2669 patients were finally eligible for investigation and were classified into five groups: 874 patients (33%) received non-maintenance six-dose iBCG (Group A; i-6), 405 (15%) received six-dose iBCG plus mBCG (Group B; i-6 + m), 1189 (44%) received non-maintenance seven−/eight-dose iBCG alone (Group C; i-7/8), 60 (2.2%) received seven−/eight-dose iBCG plus mBCG (Group D; i-7/8 + m); and 141 (5.3%) received only ≤5 BCG doses during iBCG (Group E; i ≤ 5) (Additional file 1: Fig. S1). Only 60 (5.0%) of 1249 patients receiving seven−/eight-dose iBCG were managed by subsequent mBCG.

### Surveillance

While surveillance protocol varied across institutions, patients were generally followed up using white-light cystoscopy and urinary cytology every 3 months for 2 years, then every 6 months in the third and fourth years, and then annually thereafter [2–4]. Recurrence was defined as recurrent tumors of pathologically proven urothelial carcinoma in bladder and prostatic urethra. Neither recurrence of upper urinary tract and positive result of urinary cytology without pathologically proven urothelial carcinoma was considered to be recurrence. Progression was defined as recurrent disease with invasion into the muscularis propria (≥T2), positive regional lymph nodes, and/or distant metastases.

### Statistical analysis

Clinicopathological characteristics were compared using Mann–Whitney U, chi-square, and Kruskal–Wallis tests, as appropriate. Recurrence-free survival (RFS), progression-free survival (PFS), and cancer-specific survival (CSS) were calculated from the date of administration of the initial iBCG dose. Survival rates were analyzed using the Kaplan–Meier method and compared using the log-rank test. Statistical analyses and plots were performed using GraphPad Prism version 7.00 (GraphPad Software, San Diego, CA, USA). All reported *P* values were two-sided, and statistical significance was set at *P* < 0.05.

The baseline characteristics were matched by calculating the propensity score for each patient using a multivariable logistic regression model based on covariates such as age, sex, past history of NMIBC, multiplicity, T category, presence of CIS, prostate-involving CIS, presence of divergent differentiation or variants, LVI, implementation of second TUR, and full or reduced dose at iBCG initiation. Propensity score matching (PSM) was performed using R version 4.0.0 (R Development Core Team, Vienna, Austria). One-to-one matching with a caliper width of 0.2 was applied to maintain a large sample size and balance between two groups for the following three pairs: Group A vs. B, Group B vs. C, and Group A vs. C. Standardized mean difference (SMD) was used to examine the balance of covariate distribution between the groups after PSM [15]. SMD less than 0.1 indicated that all covariates were balanced after PSM.

To evaluate the durability of the clinical benefit of mBCG, we considered the immortal time bias due to the time-dependent nature of mBCG and response to intravesical BCG, which varies from patient to patient [16]. As mBCG continuation was defined over the observation period for RFS and all-cause mortality, we performed conditional landmark analyses of RFS at 6, 12, 18, and 24 months to compare Groups A and B and Groups B and C. Patients who experienced recurrence, were censored by death, or lost to follow-up within each landmark after iBCG initiation were excluded from the landmark analyses. Kaplan–Meier curves were constructed for each treatment group to compare RFS using the log-rank test.

## Results

### Comparison of patients’ characteristics and outcomes among the BCG treatment groups

**Fig. 2:**
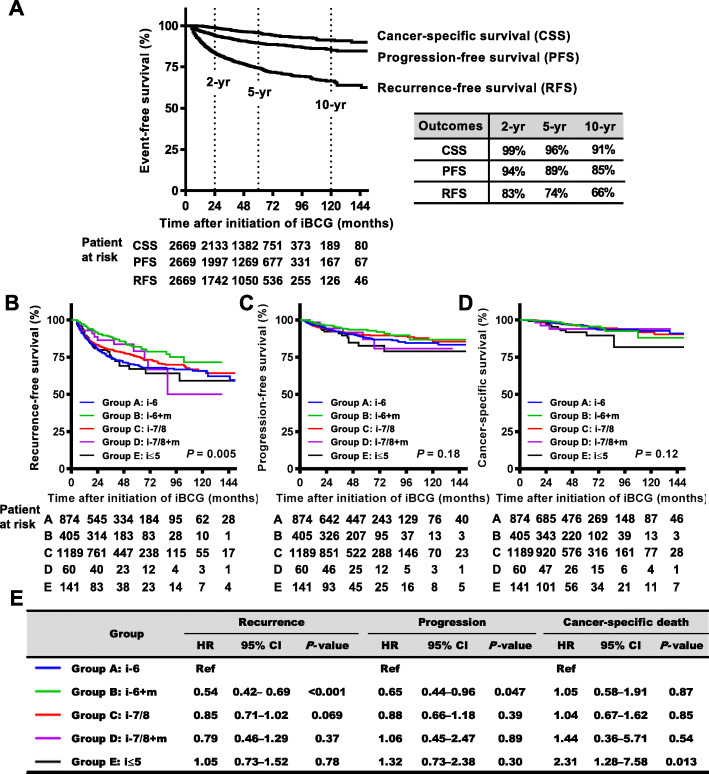
Survival curves of outcomes after iBCG according to BCG treatment patterns. **a** Recurrence-free survival (RFS), progression-free survival (PFS), and cancer-specific survival (CSS) of a total of 2669 patients treated with intravesical BCG are plotted and the estimated 2-, 5-, and 10-year survivals after initiation of induction BCG (iBCG) are shown on the right. RFS (**b**), PFS (**c**), and CSS (**d**) are plotted and compared among five groups as follows: Group **a** (i-6), non-maintenance six-dose iBCG; Group **b** (i-6 + m), six-dose iBCG plus mBCG; Group **c** (i-7/8), non-maintenance seven−/eight-dose iBCG; Group **d** (i-7/8 + m), seven−/eight-dose iBCG plus mBCG; and Group **e** (i ≤ 5), 5 or fewer BCG doses in iBCG. **e** The hazard ratios (HRs) and 95% confidence intervals (CIs) for groups **b**-**e** relative to Group A were calculated using the log-rank test and tabulated. *P* values are also listed

Table S1 (Additional file 2**)** summarizes the patient characteristics of five groups. Multiple comparisons of Groups A-D showed significant differences in age, sex, T category, and presence of CIS. As Group E included patients who were intolerant to iBCG course [17], age and dose reduction rate in iBCG were higher than those of other groups. The completion rate of planned iBCG was 93 and 83% with 6-dose iBCG and 8-dose iBCG, respectively. The reason for incompletion varied among patients, most frequently due to severe adverse events, followed by patients’ refusal of treatment, and physicians’ decision, resulting in a mean BCG dose of 7.4 doses and mean treatment duration of mBCG in Group B for 9 months. Of 405 patients in Group B, 13 (3.2%) were treated with alternative mBCG regimens such as monthly single instillations (less intensified than the standard regimen). Of 2669 patients, 585 (22%) and 241 (9.0%) experienced recurrence and progression, respectively, and 110 (4.1%) died due to bladder cancer, with a median follow-up of 48 months (interquartile range, 27–75) after iBCG initiation and 1984 patients have been followed without recurrence for 46 months (24–72). The 2−/5-year RFS and PFS rates of 2669 patients were 83%/74 and 94%/89%, respectively (Fig. 2a). To investigate the impact of BCG treatment patterns on oncological outcomes, RFS, PFS, and CSS were compared among the five groups using unmatched univariate analysis (Fig. 2b-e). Group B (i-6 + m) showed significantly longer RFS (hazard ratio [HR] 0.54, 95% confidence interval [CI] 0.42–0.69) and PFS (HR 0.65, 95% CI 0.44–0.96) than Group A (i-6). Group C (i-7/8) had a tendency toward longer RFS (HR 0.85, 95%CI 0.71–1.02) than Group A (i-6). Group C (i-7/8) had a tendency toward longer RFS (HR 0.85, 95%CI 0.71–1.02) compared to Group A (i-6). Group E showed the worst outcome regarding CSS.

### Comparison of outcomes among propensity score-matched cohorts

**Fig. 3:**
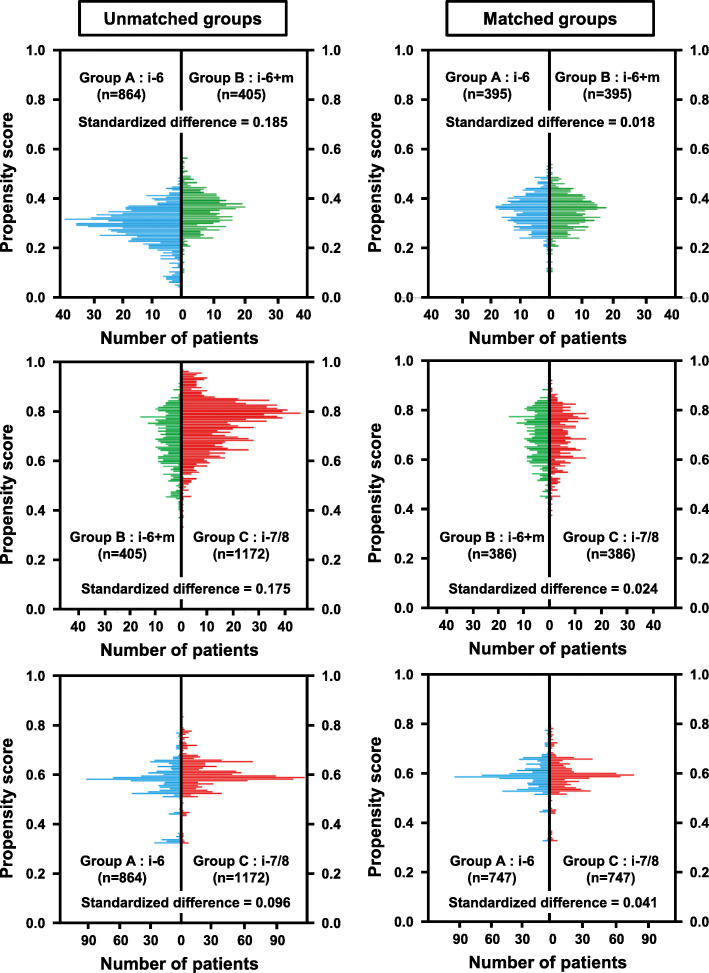
Distributions of propensity scores in the unmatched and matched groups. The propensity score was calculated for each patient using a multivariable logistic regression model based on the covariates shown in Table S2 (Additional file 3) and Table S3 (Additional file 4). Comparison of variables according to patterns of intravesical BCG treatment: after propensity score matching. One-to-one matching with a caliper width of 0.2 was applied to maintain a large sample size and balance between two groups: Group **a** vs. Group **b** (upper panels), Group **b** vs. Group **c** (middle panels), and Group **a** vs. Group **c** (lower panels)

PSM was applied to adjust for patient demographic characteristics across treatment groups and decrease the influence of possible confounding factors. Table S2 (Additional file 3) lists clinicopathologic covariates of Groups A-C and shows a comparison of three pairs before PSM. Patients with missing data in at least one covariate were excluded from PSM (complete case analysis) [18]. Several covariates showed significant differences between two groups before PSM. Figure 3 shows the distributions of propensity scores in the unmatched and matched groups. Adjustment using PSM resulted in a closely balanced distribution of the baseline covariates between two groups in each pair (Additional file 4: Table S3).

Comparisons of the three endpoints in three pairs before and after PSM are shown in Table 1. A significant benefit of mBCG following six-dose iBCG (Group B) for recurrence risk reduction was retained after PSM as compared to non-maintenance six-dose iBCG (Group A) and non-maintenance seven−/eight-dose iBCG (Group C). PFS significantly differed between unmatched Groups A and B (*P* = 0.047) but not between the matched cohorts (*P* = 0.36). Overall, there were no significant differences in the risks of progression and cancer-specific death.

**Table 1:** Outcomes among three pairs of patients with NMIBC before and after propensity score matching

Outcomes	Groups	Before propensity score matching				After propensity score matching			
		*N*	HR	95% CI	*P-*value ^#^	*N*	HR	95% CI	*P*-value ^#^
Recurrence	Group A: i-6	874	Ref			395	Ref		
	Group B: i-6+m	405	0.54	0.42– 0.69	<0.0001	395	0.59	0.43–0.81	0.001
	Group C: i-7/8	1189	Ref			386	Ref		
	Group B: i-6+m	405	0.64	0.50– 0.82	0.0016	386	0.64	0.46– 0.89	0.0074
	Group A: i-6	874	Ref			747	Ref		
	Group C: i-7/8	1189	0.85	0.71–1.02	0.069	747	0.93	0.75–1.14	0.49
Progression	Group A: i-6	874	Ref			395	Ref		
	Group B: i-6+m	405	0.65	0.44–0.96	0.047	395	0.79	0.48–1.31	0.36
	Group C: i-7/8	1189	Ref			386	Ref		
	Group B: i-6+m	405	0.73	0.50– 1.09	0.15	386	0.66	0.40– 1.10	0.11
	Group A: i-6	874	Ref			747	Ref		
	Group C: i-7/8	1189	0.88	0.66–1.18	0.39	747	0.97	0.70–1.34	0.84
Cancer-specific death	Group A: i-6	874	Ref			395	Ref		
	Group B: i-6+m	405	1.05	0.58–1.91	0.87	395	1.17	0.58–2.37	0.66
	Group C: i-7/8	1189	Ref			386	Ref		
	Group B: i-6+m	405	1.05	0.59– 1.87	0.87	386	0.80	0.41– 1.55	0.51
	Group A: i-6	874	Ref			747	Ref		
	Group C: i-7/8	1189	1.04	0.67–1.62	0.85	747	1.29	0.76– 2.17	0.32

*NMIBC* non-musle invasive bladder cancer, *BCG* Bacille de Calmette et Guérin, *i* induction, *m* meintenance, *Ref* reference (set to 1.0), *HR* hazard ratio, *CI* confidence interval, ^#^Log-rank test

### Conditional landmark analysis of RFS after iBCG

**Fig. 4:**
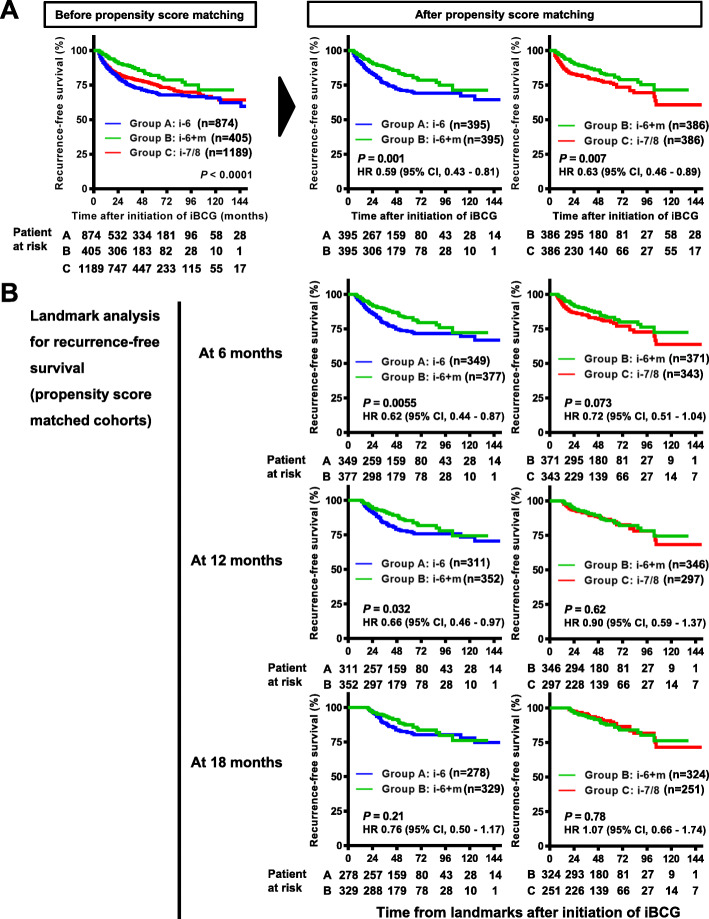
Sequential landmark analyses of recurrence among patients treated by non-maintenance induction BCG (iBCG) or iBCG plus maintenance BCG (mBCG). **a** Recurrence-free survival curves (RFS) after iBCG are plotted and compared using the log-rank test among three groups as follows: Group **a** (i-6), non-maintenance six-dose iBCG; Group **b** (i-6 + m), six-dose iBCG plus mBCG; and Group **c** (i-7/8), non-maintenance seven−/eight-dose iBCG. After propensity score matching, RFS curves were compared between groups **a** and **b** and between groups **b** and **c**. **b** Landmark RFS curves at 6, 12, and 18 months for recurrence among patients treated with non-maintenance iBCG or iBCG plus mBCG were plotted and compared between matched groups **a** and **b** (left panels) and between matched groups **b** and **c** (right panels). The *P* values, hazard ratios (HRs) and 95% confidence intervals (CIs) are shown in the figures

RFS curves of unmatched groups A-C showed a clear benefit of subsequent mBCG compared to non-maintenance groups (Fig. 4a, left panel). This benefit remained even after PSM (Fig. 4a, right panels). To exclude the immortal time bias and evaluate the durability of iBCG and mBCG, we performed sequential landmark analyses of the matched cohorts at multiple time points. Landmark analyses at 6 and 12 months showed a benefit of mBCG following six-dose iBCG compared to non-maintenance six-dose iBCG, whereas landmark analysis at 18 and 24 months showed no significant difference between the two groups (Fig. 4b and Additional file 5: Fig. S2, left panels). Contrarily, no significant difference between mBCG following six-dose iBCG and non-maintenance seven−/eight-dose iBCG was observed in landmark Kaplan–Meier analyses at 12, 18, and 24 months (Fig. 4b and Additional file 5: Fig. S2, right panels). Landmark Kaplan–Meier analyses at 6 months showed a tendency of better outcome in mBCG following six-dose iBCG compared to non-maintenance seven−/eight-dose iBCG (*P* = 0.073; HR 0.72, 95%CI 0.51–1.04).

## Discussion

We compared clinical outcomes among different patterns of BCG treatment using a large real-world dataset derived from more than 2600 patients with high- or highest-risk NMIBC. This study aimed to evaluate whether non-maintenance eight-dose iBCG was comparable to six-dose iBCG plus mBCG. As our database was based on patients’ data that were derived from many sources associated with outcomes in a heterogeneous population, PSM was applied to adjust the pre-iBCG baseline characteristics on controlled background covariates to minimize biases (Fig. 3, Table S2, and Table S3).

The RFS curves of the matched cohorts of Group B (i-6 + m) and Group C (i-7/8) showed that six-dose iBCG plus mBCG yielded better outcomes compared to non-maintenance seven−/eight-dose iBCG in the non-landmark analysis (Fig. 4a). Survival analyses are frequently subject to immortal time bias, also known as guarantee-time bias [19]. The time-dependent nature of mBCG continuation and response to intravesical BCG, which varies from patient to patient, could result in immortal time bias. Landmark analysis is a valid and widely accepted method of reducing bias in survival estimation [16]. Patients who experienced recurrence or censoring before the landmark were excluded from the subsequent landmark analysis. To determine the clinical benefit of seven−/eight-dose iBCG over a longer period after iBCG completion, landmark time points (6, 12, 18, and 24 months) were selected based on the timing of BCG dosing in mBCG [10]. Kaplan–Meier plots of RFS at the three landmarks showed no significant difference between matched cohort pairs of non-maintenance seven−/eight-dose iBCG and six-dose iBCG plus mBCG at 12, 18, and 24 months (Fig. 4b and Fig. S2). Contrarily, marked differences between six-dose iBCG and six-dose iBCG plus mBCG were observed in the 6- and 12-month landmark analyses. These findings suggested that seven−/eight-dose iBCG might exert a durable effect comparable to that of six-dose iBCG plus mBCG but not among some patients, who experienced no tumor recurrence 4–6 months after iBCG initiation. There seemed to be an aggressive subset for which non-maintenance iBCG was insufficient to control early relapse within 6 months.

Undoubtedly, BCG is the first-line drug for NMIBC. However, NMIBC management currently faces a chronic dearth of BCG [9, 20, 21]. Appropriate management of bladder cancer is a serious issue in low-income countries, which cannot afford to import enough supply of BCG [22]. There is an urgent need to re-optimize the use of scarce BCG vials. As key international guidelines are constantly updated, significant concerns and efforts in the BCG shortage era have been discussed in recent versions [23]. The Bladder Cancer Advocacy Network (BCAN) released a joint statement of different urological societies and general recommendations to face BCG shortage (https://bcan.org/bladder-cancer-bcg-shortage-webinar/). For high-risk NMIBC, mBCG can be shortened to 1 year for Ta high-grade tumors, and one-third of the BCG dose can be considered for iBCG and mBCG. The comparative efficacy of reduced BCG dose (minimum, one-third) was supported by findings of randomized controlled trials [24–27]. The BCAN states that alternatives include chemotherapeutic agents for induction and maintenance for up to 1 year, and immediate radical cystectomy is recommended for patients with highest-risk NMIBC, such as T1 high-grade tumors with concomitant CIS. In reality, some physicians may hesitate to decide the mBCG treatment and some institutions might restrict mBCG due to BCG shortage. Our finding could not clear benefit of additional two BCG doses in induction phase statistically by the direct comparison between non-maintenance eight-dose iBCG (Group A) and non-maintenance six-dose iBCG (Group C). However, additional two or three doses in induction phase could be a next game changer.

One of the pivotal randomized controlled trials from EORTC-GU cancers group demonstrated that intermediate-risk NMIBC patients treated with 15 doses of BCG for 1 year did not benefit from an additional 2 years of mBCG [27]. However, in the high-risk NMIBC patients, 3-year mBCG was associated with a reduction only in bladder recurrence, not in progression or survival. Our cohort did not include the intermediate-risk NMIBC. Here, we propose a novel possibility of non-maintenance eight-dose iBCG as an alternative remedy in the high- or highest-risk NMIBC for BCG shortage. Our previous study suggested that eight-dose iBCG could induce higher levels of systemic and local immune response than six-dose iBCG, supporting the rationale of adding two doses to the standard six-dose regimen [13]. Regarding the use of BCG vials, mBCG requires 6–21 additional BCG doses after iBCG [5]. Replacing iBCG plus mBCG with non-maintenance eight-dose iBCG could save substantial amounts of BCG. Moreover, physicians should consider persistent deterioration in functional outcomes related to mBCG. Serretta et al. evaluated compliance in 411 patients undergoing 1-year mBCG, demonstrating that the largest reason for stopping mBCG was mild discomfort and deterioration in quality of social life in 55 (59.1%) out of 93 patients [28]. In our matched cohort analysis, subsequent mBCG was unable to decrease the risks of progression and cancer-specific death compared to non-maintenance iBCG (Table 1**)**. We did not perform additional landmark analyses of PFS and CSS in this study. As most intravesical recurrent tumors after iBCG completion were not life-threatening, the negative effect on possible side effects and high cost of mBCG may outweigh its oncological benefit. Landmark analysis showed durable benefit of eight-dose iBCG for patients who had no recurrence within 12 months. Taken together, non-maintenance eight-dose iBCG may be an alternative for high-risk NMIBC in selected patients. However, there is a need to for clinicians consider that early recurrence of high-grade NMIBC was labelled as ‘BCG-unresponsive’, which should be recommended for immediate cystectomy [2].

This study had several limitations. First, its retrospective nature had an inherent potential for selection bias; for example, the criteria, dose, and schedule of BCG treatment depended on the institutional protocol and physician’s discretion. The cohort was derived from multiple institutions, which could introduce inconsistencies in surgical skills, clinical interpretations, and pathological diagnoses. We enrolled the patients who were treated between 2000 and 2019. During two decades, the treatment strategy, modality, and surgical skill has changed over time, that could influence the outcomes. Second, patient grouping according to BCG treatment pattern in this study may be inaccurate because initial intention-to-treat (iBCG alone or iBCG plus mBCG) data were unavailable. Third, the oncological effect derived from BCG dose reduction of iBCG and during mBCG was not fully evaluated. Fourth, the database included patients treated with the Connaught strain, which is currently unavailable. Moreover, the database did not include any patients treated with Tice and other strains that are used widely in Europe, the United States, and Canada.

Although many clinical trials are ongoing to evaluate alternative drugs such as immune checkpoint inhibitors and novel anticancer reagents for BCG-naïve high-risk NMIBC and BCG-unresponsive NMIBC [21], we still need to await approval and validation of upcoming strategies. Even if approved, we expect that those drugs will be too expensive for widespread use among all patients. Non-maintenance eight-dose iBCG was inferior to six-dose iBCG plus mBCG. Although six-dose iBCG plus mBCG should be considered the standard of care for high-risk NMIBC, our study showed that non-maintenance eight-dose iBCG could be a potential alternative remedy in the BCG shortage era for the first time. Further investigation is warranted to confirm the real clinical value of non-maintenance eight-dose iBCG.

## Supplementary Information

**Additional file 1: Fig. S1**. Distribution of doses of induction BCG (iBCG). A total of 2669 patients were stratified according to the number of BCG doses administered in iBCG. Most of the patients classified in the iBCG ≤5 group (*n* = 141) were BCG-intolerant patients who were unable to tolerate at least one full iBCG course. While six-dose iBCG is commonly used in the United States and Europe, eight-dose iBCG is frequently and traditionally used in Japan. In addition, maintenance BCG (mBCG) has not been recognized as a standard treatment option in Japan. The proportions of patients treated with mBCG were 31.7% (green bar, 405 of 1279) and 5.0% (purple bar, 60 of 1249) among patients undergoing six-dose iBCG and seven−/eight-dose iBCG, respectively.

**Additional file 2: Table S1**. Additional file 2: Table S1. Comparison of variables of patients with NMIBC according to the BCG treatment patterns.

**Additional file 3: Table S2**. Comparison of variables according to patterns of intravesical BCG treatment: before propensity score matching.

**Additional file 4: Table S3**. Comparison of variables according to patterns of intravesical BCG treatment: after propensity score matching.

**Additional file 5: Fig. S2**. Landmark analysis of recurrence among patients treated with non-maintenance induction BCG (iBCG) or iBCG plus maintenance BCG (mBCG) at 24 months. Landmark RFS at 6, 12, and 18 months among patients treated with non-maintenance iBCG or iBCG plus mBCG are plotted and compared between matched groups A and B (left panel) and between matched groups B and C (right panel). The *P* values, hazard ratios (HRs), and 95% confidence intervals (CIs) are shown in the figures.

## Data Availability

The datasets used and/or analyzed during the current study are available from the corresponding author on reasonable request.

## Abbreviations

BCG: Bacillus calmette–guérin
CIS: Carcinoma in situ
CSS: Cancer-specific survival
EAU: European association of urology
HR: Hazard ratio
iBCG: Induction BCG
LVI: lymphovascular involvement
mBCG: Maintenance BCG
NMIBC: Non-muscle-invasive bladder cancer
PFS: Progression-free survival
PSM: Propensity score matching
RFS: Recurrence-free survival
SMD: Standardized mean difference
TURBT: Transurethral resection of bladder tumor
