# Supplementary figures and images for: Non-maintenance intravesical Bacillus Calmette–Guérin induction therapy with eight doses in patients with high- or highest-risk non-muscle invasive bladder cancer: a retrospective non-randomized comparative study
Source: BMC Cancer. 2021 Mar 11;21:266. doi: 10.1186/s12885-021-07966-7 (PMC7948348; doi:10.1186/s12885-021-07966-7)

## Slide 1
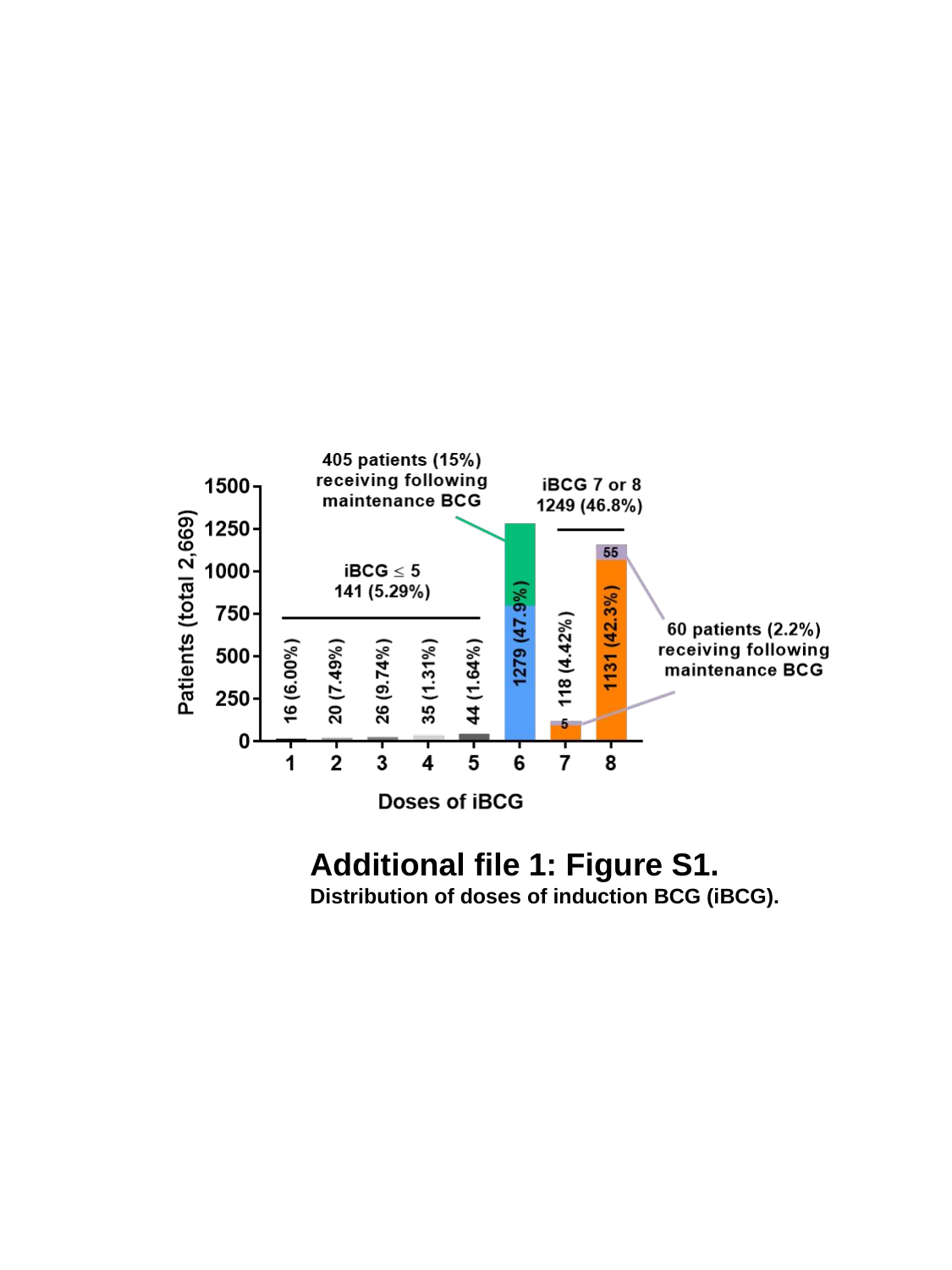

Additional file 1: Figure S1.
Distribution of doses of induction BCG (iBCG).

Supplement: Supplementary file 1 — Additional file 1: Fig. S1. Distribution of doses of induction BCG (iBCG). A total of 2669 patients were stratified according to the number of BCG doses administered in iBCG. Most of the patients classified in the iBCG ≤5 group (n = 141) were BCG-intolerant patients who were unable to tolerate at least one full iBCG course. While six-dose iBCG is commonly used in the United States and Europe, eight-dose iBCG is frequently and traditionally used in Japan. In addition, maintenance BCG (mBCG) has not been recognized as a standard treatment option in Japan. The proportions of patients treated with mBCG were 31.7% (green bar, 405 of 1279) and 5.0% (purple bar, 60 of 1249) among patients undergoing six-dose iBCG and seven−/eight-dose iBCG, respectively. [file 12885_2021_7966_MOESM1_ESM.pptx]
