# Supplementary material for: Non-maintenance intravesical Bacillus Calmette–Guérin induction therapy with eight doses in patients with high- or highest-risk non-muscle invasive bladder cancer: a retrospective non-randomized comparative study
Source: BMC Cancer. 2021 Mar 11;21:266. doi: 10.1186/s12885-021-07966-7 (PMC7948348; doi:10.1186/s12885-021-07966-7)
Supplement: Supplementary file 2 — Additional file 2: Table S1. Additional file 2: Table S1. Comparison of variables of patients with NMIBC according to the BCG treatment patterns. [file 12885_2021_7966_MOESM2_ESM.docx]

| **Additional file 2: Table S1. Comparison of variables of patients with NMIBC according to the BCG treatment patterns** | | | | | | | | | | | | |
| --- | --- | --- | --- | --- | --- | --- | --- | --- | --- | --- | --- | --- |
| **Variables** |  | **Total** |  | **iBCG 6 doses** | |  | **iBCG 7/8 doses** | |  | **Group E iBCG ≤ 5 doses** | **Multiple comparison of four groups (Groups A to D)** | **Comparison between Group E and other goups (Groups A−D)** |
|  |  |  |  | **Group A iBCG alone** | **Group B iBCG + mBCG** |  | **Group C iBCG alone** | **Group D iBCG + mBCG** |  |  |  |  |
| **N** |  | **2669 (100%)** |  | **874 (33%)** | **405 (15%)** |  | **1189 (44%)** | **60 (2.2%)** |  | **141 (5.3%)** | **-** | **-** |
| **Age, mean ± SD** |  | **71.2 ± 9.3** |  | **71.4 ± 9.3** | **69.6 ± 9.9** |  | **71.3 ± 9.3** | **71.8 ± 9.9** |  | **73.7 ± 7.9** | **0.019 #** | **0.002 ####** |
| **Sex** |  |  |  |  |  |  |  |  |  |  | **0.003 ##** | **0.022 ##** |
| **Male** |  | **2197 (82%)** |  | **725 (76%)** | **341 (83%)** |  | **986 (84%)** | **39 (65%)** |  | **106 (75%)** |  |  |
| **Female** |  | **472 (18%)** |  | **149 (24%)** | **64 (17%)** |  | **203 (16%)** | **21 (35%)** |  | **35 (25%)** |  |  |
| **ECOG-PS** |  |  |  |  |  |  |  |  |  |  | **0.002 ###** | **0.080 ###** |
| **0** |  | **2264 (85%)** |  | **714 (82%)** | **365 (90%)** |  | **1015 (85%)** | **54 (90%)** |  | **116 (88%)** |  |  |
| **1** |  | **220 (8.2%)** |  | **67 (7.7%)** | **15 (3.7%)** |  | **122 (10%)** | **5 (8.3%)** |  | **11 (7.8%)** |  |  |
| **2** |  | **43 (1.6%)** |  | **16 (1.8%)** | **1 (0.3%)** |  | **25 (2.1%)** | **0 (0%)** |  | **1 (0.7%)** |  |  |
| **3-4** |  | **16 (0.6%)** |  | **5 (0.6%)** | **0 (0%)** |  | **8 (0.7%)** | **0 (0%)** |  | **3 (2.1%)** |  |  |
| **Unknown** |  | **126 (4.7%)** |  | **72 (8.2%)** | **24 (5.9%)** |  | **19 (1.6%)** | **1 (1.6%)** |  | **10 (7.8%)** |  |  |
| **Past history of NMIBC** |  |  |  |  |  |  |  |  |  |  | **0.27 ##** | **0.63 ##** |
| **Primary case** |  | **2125 (80%)** |  | **684 (78%)** | **336 (83%)** |  | **946 (80%)** | **49 (82%)** |  | **110 (78%)** |  |  |
| **Recurrent case** |  | **544 (20%)** |  | **190 (22%)** | **69 (17%)** |  | **243 (20%)** | **11 (18%)** |  | **31 (22%)** |  |  |
| **Multiplicity** |  |  |  |  |  |  |  |  |  |  | **0.97 ###** | **0.045 ###** |
| **Single** |  | **799 (30%)** |  | **248 (28%)** | **125 (31%)** |  | **355 (30%)** | **18 (30%)** |  | **53 (38%)** |  |  |
| **Multiple** |  | **1781 (67%)** |  | **566 (65%)** | **275 (68%)** |  | **818 (69%)** | **38 (63%)** |  | **84 (60%)** |  |  |
| **Unknown** |  | **89 (3.3%)** |  | **60 (6.9%)** | **5 (1.2%)** |  | **16 (1.4%)** | **4 (6.7%)** |  | **4 (2.8%)** |  |  |
| **Tumor size** |  |  |  |  |  |  |  |  |  |  | **0.34 ###** | **0.98 ###** |
| **< 3 cm** |  | **1562 (59%)** |  | **502 (58%)** | **267 (66%)** |  | **676 (57%)** | **28 (47%)** |  | **86 (61%)** |  |  |
| **≥ 3 cm** |  | **434 (16%)** |  | **139 (16%)** | **81 (20%)** |  | **177 (15%)** | **13 (22%)** |  | **24 (17%)** |  |  |
| **Unknown** |  | **673 (25%)** |  | **230 (26%)** | **57 (14%)** |  | **336 (28%)** | **19 (32%)** |  | **31 (22%)** |  |  |
| **T category** |  |  |  |  |  |  |  |  |  |  | **0.003 ##** | **0.008 ##** |
| **Ta** |  | **623 (23%)** |  | **178 (20%)** | **119 (29%)** |  | **293 (25%)** | **13 (22%)** |  | **20 (15%)** |  |  |
| **T1** |  | **1443 (54%)** |  | **501 (57%)** | **213 (52%)** |  | **604 (51%)** | **32 (53%)** |  | **93 (65%)** |  |  |
| **Pure Tis** |  | **603 (23%)** |  | **195 (22%)** | **73 (18%)** |  | **292 (25%)** | **15 (25%)** |  | **28 (19%)** |  |  |
| **Tumor grade (WHO 2004)** | |  |  |  |  |  |  |  |  |  | **0.18 ##** | **0.76 ##** |
| **Low grade** |  | **67 (2.5%)** |  | **14 (1.6%)** | **11 (2.7%)** |  | **37 (3.1%)** | **2 (3.3%)** |  | **3 (2.1%)** |  |  |
| **High grade** |  | **2602 (97%)** |  | **860 (98%)** | **394 (97%)** |  | **1152 (97%)** | **58 (97%)** |  | **138 (98%)** |  |  |
| **CIS** |  |  |  |  |  |  |  |  |  |  | **< 0.0001 ##** | **0.038 ##** |
| **No** |  | **1213 (45%)** |  | **432 (49%)** | **229 (57%)** |  | **453 (38%)** | **23 (38%)** |  | **76 (54%)** |  |  |
| **Yes** |  | **1456 (55%)** |  | **442 (51%)** | **176 (43%)** |  | **736 (62%)** | **37 (62%)** |  | **65 (46%)** |  |  |
| **Prostate-involving CIS** |  |  |  |  |  |  |  |  |  |  | **0.67 ##** | **0.22 ##** |
| **No** |  | **2613 (98%)** |  | **855 (98%)** | **398 (98%)** |  | **1164 (98%)** | **60 (100%)** |  | **136 (96%)** |  |  |
| **Yes** |  | **56 (2.1%)** |  | **19 (2.2%)** | **7 (1.7%)** |  | **25 (2.1%)** | **0 (0%)** |  | **5 (3.5%)** |  |  |
| **Divergent differentiation**  **or variants** |  |  |  |  |  |  |  |  |  |  | **0.08 ##** | **0.60 ##** |
| **No** |  | **2572 (96%)** |  | **849 (97%)** | **395 (98%)** |  | **1134 (95%)** | **57 (95%)** |  | **137 (97%)** |  |  |
| **Yes** |  | **97 (3.6%)** |  | **25 (2.9%)** | **10 (2.5%)** |  | **55 (4.6%)** | **3 (5.0%)** |  | **4 (2.8%)** |  |  |
| **LVI** |  |  |  |  |  |  |  |  |  |  | **0.0013 ##** | **0.81 ##** |
| **No** |  | **2547 (95%)** |  | **834 (95%)** | **401 (99%)** |  | **1122 (94%)** | **56 (93%)** |  | **134 (95%)** |  |  |
| **Yes** |  | **122 (4.5%)** |  | **40 (4.6%)** | **4 (1.0%)** |  | **67 (5.6%)** | **4 (6.7%)** |  | **7 (4.9%)** |  |  |
| **Second TUR** |  |  |  |  |  |  |  |  |  |  | **< 0.0001 ##** | **0.14 ##** |
| **No** |  | **1709 (64%)** |  | **613 (70%)** | **264 (65%)** |  | **719 (60%)** | **31 (52%)** |  | **82 (58%)** |  |  |
| **Yes** |  | **960 (36%)** |  | **261 (30%)** | **141 (35%)** |  | **470 (40%)** | **29 (48%)** |  | **59 (42%)** |  |  |
| **BCG strain** |  |  |  |  |  |  |  |  |  |  | **0.0045 ###** | **0.28 ###** |
| **Tokyo 172** |  | **2089 (78%)** |  | **663 (76%)** | **301 (74%)** |  | **962 (81%)** | **48 (80%)** |  | **115 (82%)** |  |  |
| **Connaught** |  | **572 (21%)** |  | **210 (24%)** | **104 (26%)** |  | **221 (19%)** | **12 (20%)** |  | **25 (18%)** |  |  |
| **Unknown** |  | **8 (0.3%)** |  | **1 (0.1%)** | **0 (0.0%)** |  | **6 (0.5%)** | **0 (0%)** |  | **1 (0.7%)** |  |  |
| **Dose reduction in iBCG** |  |  |  |  |  |  |  |  |  |  | **< 0.0001 ##** | **0.006 ##** |
| **Full dose** |  | **2224 (83%)** |  | **689 (79%)** | **329 (81%)** |  | **1053 (89%)** | **48 (80%)** |  | **105 (74%)** |  |  |
| **Reduced dose** |  | **430 (16%)** |  | **181 (21%)** | **76 (19%)** |  | **128 (11%)** | **11 (18%)** |  | **34 (24%)** |  |  |
| **Unknown** |  | **15 (0.6%)** |  | **4 (0.5%)** | **0 (0%)** |  | **8 (0.7%)** | **1 (1.7%)** |  | **2 (1.4%)** |  |  |
| **Total doses of maintenance BCG** | | |  |  |  |  |  |  |  |  | **0.97 #** | **-** |
| **Mean ± SD** |  | **7.3 ± 6.7** |  | **-** | **7.4 ± 7.0** |  | **-** | **6.9 ± 5.0** |  | **-** |  |  |
| **Median, range** |  | **6 (2-39)** |  | **-** | **6 (2-39)** |  | **-** | **6 (2-24)** |  | **-** |  |  |
| **NMIBC, non-muscle invasive bladder cancer; BCG, ; iBCG, induction BCG; mBCG, meintenance BCG; SD, standard deviation; TURBT, transurethral resection of the bladder tumor; WHO, the World Health Organization; CIS, carcinoma *in situ*; LVI, lymphovascular invasion; TUR, transurethral resection; # Kruskal-Wallis test; ## Chi-square test; ### Chi-square test excluding missing data; #### Mann–Whitney U test** | | | | | | | | | | | | |
