# Supplementary material for: Non-maintenance intravesical Bacillus Calmette–Guérin induction therapy with eight doses in patients with high- or highest-risk non-muscle invasive bladder cancer: a retrospective non-randomized comparative study
Source: BMC Cancer. 2021 Mar 11;21:266. doi: 10.1186/s12885-021-07966-7 (PMC7948348; doi:10.1186/s12885-021-07966-7)
Supplement: Supplementary file 3 — Additional file 3: Table S2. Comparison of variables according to patterns of intravesical BCG treatment: before propensity score matching. [file 12885_2021_7966_MOESM3_ESM.docx]

| **Additional file 3: Table S2. Comparison of variables according to patterns of intravesical BCG treatment: before propensity score matching** | | | | | | | | | | | | | | | |  |
| --- | --- | --- | --- | --- | --- | --- | --- | --- | --- | --- | --- | --- | --- | --- | --- | --- |
| **Variables** |  | **Group A iBCG-6 alone** | **Group B iBCG-6 + mBCG** | ***P* value** | **SMD** |  | **Group B iBCG-6 + mBCG** | **Group C iBCG-7/8 alone** | **P value** | **SMD** |  | **Group A iBCG-6 alone** | **Group C iBCG-7/8 alone** | **P value** | **SMD** |  |
| **N** |  | **864** | **405** | **-** | **-** |  | **405** | **1172** | **-** | **-** |  | **864** | **1172** | **-** | **-** |  |
| **Age, mean ± SD** |  | **71.4 ± 9.3** | **69.6 ± 9.9** | **0.002** | **0.18** |  | **69.6 ± 9.9** | **71.3 ± 9.3** | **0.002** | **0.17** |  | **71.4 ± 9.3** | **71.3 ± 9.3** | **0.83** | **0.01** |  |
| **Sex** |  |  |  | **0.69** | **0.03** |  |  |  | **0.59** | **0.04** |  |  |  | **0.91** | **0.01** |  |
| **Male** |  | **719 (83%)** | **341 (84%)** |  |  |  | **341 (84%)** | **972 (83%)** |  |  |  | **719 (83%)** | **972 (83%)** |  |  |  |
| **Female** |  | **145 (17%)** | **64 (16%)** |  |  |  | **64 (16%)** | **200 (17%)** |  |  |  | **145 (17%)** | **200 (17%)** |  |  |  |
| **Past history of NMIBC** |  |  |  | **0.052** | **0.12** |  |  |  | **0.15** | **0.08** |  |  |  | **0.47** | **0.03** |  |
| **Primary case** |  | **676 (78%)** | **336 (83%)** |  |  |  | **336 (83%)** | **933 (80%)** |  |  |  | **676 (78%)** | **933 (80%)** |  |  |  |
| **Recurrent case** |  | **188 (22%)** | **69 (17%)** |  |  |  | **69 (17%)** | **239 (20%)** |  |  |  | **188 (22%)** | **239 (20%)** |  |  |  |
| **Multiplicity** |  |  |  | **0.84** | **0.01** |  |  |  | **0.75** | **0.02** |  |  |  | **0.88** |  |  |
| **Single** |  | **248 (31%)** | **125 (31%)** |  |  |  | **125 (31%)** | **351 (30%)** |  |  |  | **248 (31%)** | **351 (30%)** |  |  |  |
| **Multiple** |  | **561 (69%)** | **275 (68%)** |  |  |  | **275 (68%)** | **808 (70%)** |  |  |  | **561 (69%)** | **808 (70%)** |  |  |  |
| **T category** |  |  |  | **0.002** |  |  |  |  | **0.025** |  |  |  |  | **0.022** |  |  |
| **Ta** |  | **178 (21%)** | **119 (29%)** |  | **0.20** |  | **119 (29%)** | **291 (25%)** |  | **0.11** |  | **178 (21%)** | **291 (25%)** |  | **0.11** |  |
| **T1** |  | **493 (57%)** | **213 (53%)** |  | **0.09** |  | **213 (53%)** | **600 (51%)** |  | **0.02** |  | **493 (57%)** | **600 (51%)** |  | **0.12** |  |
| **Pure Tis** |  | **193 (22%)** | **73 (18%)** |  | **0.11** |  | **73 (18%)** | **281 (24%)** |  | **0.14** |  | **193 (22%)** | **281 (24%)** |  | **0.04** |  |
| **Tumor grade (WHO 2004)** |  |  |  | **0.21** | **0.09** |  |  |  | **0.87** | **0.02** |  |  |  | **0.035** | **0.11** |  |
| **Low grade** |  | **15 (1.7%)** | **12 (3.0%)** |  |  |  | **12 (3.0%)** | **39 (3.3%)** |  |  |  | **15 (1.7%)** | **39 (3.3%)** |  |  |  |
| **High grade** |  | **849 (98%)** | **393 (97%)** |  |  |  | **393 (97%)** | **1133 (97%)** |  |  |  | **849 (98%)** | **1133 (97%)** |  |  |  |
| **CIS** |  |  |  | **0.022** | **0.14** |  |  |  | **<0.001** | **0.37** |  |  |  | **<0.001** | **0.22** |  |
| **No** |  | **430 (50%)** | **230 (57%)** |  |  |  | **230 (57%)** | **454 (39%)** |  |  |  | **430 (50%)** | **454 (39%)** |  |  |  |
| **Yes** |  | **434 (51%)** | **175 (43%)** |  |  |  | **175 (43%)** | **718 (61%)** |  |  |  | **434 (50%)** | **718 (61%)** |  |  |  |
| **Prostate-involving CIS** |  |  |  | **1.00** | **0.02** |  |  |  | **0.84** | **0.03** |  |  |  | **0.88** | **0.01** |  |
| **No** |  | **855 (98%)** | **398 (98%)** |  |  |  | **398 (98%)** | **1147 (98%)** |  |  |  | **847 (98%)** | **1147 (98%)** |  |  |  |
| **Yes** |  | **19 (2.2%)** | **7 (1.7%)** |  |  |  | **7 (1.7%)** | **25 (2.1%)** |  |  |  | **17 (2.0%)** | **25 (2.1%)** |  |  |  |
| **Divergent differentiation**  **or variants** |  |  |  | **0.72** | **0.03** |  |  |  | **0.059** | **0.12** |  |  |  | **0.049** | **0.09** |  |
| **No** |  | **839 (97%)** | **395 (98%)** |  |  |  | **395 (98%)** | **1117 (95%)** |  |  |  | **839 (97%)** | **1117 (95%)** |  |  |  |
| **Yes** |  | **25 (2.9%)** | **10 (2.5%)** |  |  |  | **10 (2.5%)** | **55 (4.7%)** |  |  |  | **25 (2.9%)** | **55 (4.7%)** |  |  |  |
| **LVI** |  |  |  | **<0.001** | **0.22** |  |  |  | **<0.001** | **0.26** |  |  |  | **0.32** | **0.05** |  |
| **No** |  | **834 (95%)** | **401 (99%)** |  |  |  | **401 (99%)** | **1105 (94%)** |  |  |  | **824 (95%)** | **1105 (94%)** |  |  |  |
| **Yes** |  | **40 (4.6%)** | **4 (1.0%)** |  |  |  | **4 (1.0%)** | **67 (5.7%)** |  |  |  | **40 (4.6%)** | **67 (5.7%)** |  |  |  |
| **Second TUR** |  |  |  | **0.092** | **0.10** |  |  |  | **0.067** | **0.11** |  |  |  | **<0.001** | **0.22** |  |
| **No** |  | **605 (70%)** | **264 (65%)** |  |  |  | **264 (65%)** | **702 (60%)** |  |  |  | **605 (70%)** | **702 (60%)** |  |  |  |
| **Yes** |  | **259 (30%)** | **141 (35%)** |  |  |  | **141 (35%)** | **470 (40%)** |  |  |  | **259 (30%)** | **470 (40%)** |  |  |  |
| **BCG dose in iBCG** |  |  |  | **0.50** | **0.05** |  |  |  | **<0.001** | **0.23** |  |  |  | **<0.001** | **0.27** |  |
| **Full dose** |  | **686 (79%)** | **329 (81%)** |  |  |  | **329 (81%)** | **1045 (89%)** |  |  |  | **686 (79%)** | **1045 (89%)** |  |  |  |
| **Reduced dose** |  | **178 (21%)** | **76 (19%)** |  |  |  | **76 (19%)** | **127 (11%)** |  |  |  | **178 (21%)** | **127 (11%)** |  |  |  |
| **NMIBC, non-muscle invasive bladder cancer; BCG, ; iBCG, induction BCG; mBCG, meintenance BCG; SMD, Standardized mean difference; SD, standard deveation; TURBT, transurethral resection of the bladder tumor; WHO, the World Health Organization; CIS, carcinoma *in situ*; LVI, lymphovascular invasion; TUR, transurethral resection** | | | | | | | | | | | | | | | |  |
|  |  |  |  |  |  |  |  |  |  |  |  |  |  |  |  |  |
