# Supplementary material for: Non-maintenance intravesical Bacillus Calmette–Guérin induction therapy with eight doses in patients with high- or highest-risk non-muscle invasive bladder cancer: a retrospective non-randomized comparative study
Source: BMC Cancer. 2021 Mar 11;21:266. doi: 10.1186/s12885-021-07966-7 (PMC7948348; doi:10.1186/s12885-021-07966-7)
Supplement: Supplementary file 4 — Additional file 4: Table S3. Comparison of variables according to patterns of intravesical BCG treatment: after propensity score matching. [file 12885_2021_7966_MOESM4_ESM.docx]

| **Additional file 4: Table S3. Comparison of variables according to patterns of intravesical BCG treatment: after propensity score matching** | | | | | | | | | | | | | | | |
| --- | --- | --- | --- | --- | --- | --- | --- | --- | --- | --- | --- | --- | --- | --- | --- |
| **Variables** |  | **Group A iBCG-6 alone** | **Group B iBCG-6 + mBCG** | ***P* value** | **SMD** |  | **Group B iBCG-6 + mBCG** | **Group C iBCG-7/8 alone** | **P value** | **SMD** |  | **Group A iBCG-6 alone** | **Group C iBCG-7/8 alone** | **P value** | **SMD** |
| **N** |  | **395** | **395** | **-** | **-** |  | **386** | **386** | **-** | **-** |  | **747** | **747** | **-** | **-** |
| **Age, mean ± SD** |  | **69.7 ± 9.8** | **69.9 ± 9.6** | **0.81** | **0.02** |  | **69.7 ± 9.8** | **69.9 ± 9.6** | **0.74** | **0.02** |  | **71.4 ± 9.3** | **71.8 ± 9.2** | **0.42** | **0.01** |
| **Sex** |  |  |  | **0.48** | **0.06** |  |  |  | **0.84** | **0.02** |  |  |  | **0.78** | **0.02** |
| **Male** |  | **342 (87%)** | **334 (85%)** |  |  |  | **324 (84%)** | **327 (85%)** |  |  |  | **629 (84%)** | **624 (84%)** |  |  |
| **Female** |  | **53 (13%)** | **64 (15%)** |  |  |  | **62 (16%)** | **59 (15%)** |  |  |  | **118 (16%)** | **123 (17%)** |  |  |
| **Past history of NMIBC** |  |  |  | **0.85** | **0.02** |  |  |  | **0.92** | **0.01** |  |  |  | **0.35** | **0.05** |
| **Primary case** |  | **327 (83%)** | **330 (84%)** |  |  |  | **322 (83%)** | **320 (83%)** |  |  |  | **154 (21%)** | **170 (23%)** |  |  |
| **Recurrent case** |  | **68 (17%)** | **65 (16%)** |  |  |  | **64 (17%)** | **66 (17%)** |  |  |  | **732 (98%)** | **732 (98%)** |  |  |
| **Multiplicity** |  |  |  | **0.84** | **0.03** |  |  |  | **0.49** | **0.06** |  |  |  | **0.91** | **0.01** |
| **Single** |  | **248 (31%)** | **125 (31%)** |  |  |  | **120 (31%)** | **130 (34%)** |  |  |  | **225 (30%)** | **228 (31%)** |  |  |
| **Multiple** |  | **561 (69%)** | **275 (68%)** |  |  |  | **266 (69%)** | **256 (66%)** |  |  |  | **522 (70%)** | **519 (70%)** |  |  |
| **T category** |  |  |  | **0.20** |  |  |  |  | **0.11** |  |  |  |  | **0.14** |  |
| **Ta** |  | **103 (26%)** | **117 (30%)** |  | **0.08** |  | **117 (30%)** | **92 (24%)** |  | **0.15** |  | **171 (23%)** | **157 (21%)** |  | **0.05** |
| **T1** |  | **232 (59%)** | **207 (52%)** |  | **0.13** |  | **198 (51%)** | **209 (54%)** |  | **0.06** |  | **407 (55%)** | **388 (52%)** |  | **0.05** |
| **Pure Tis** |  | **60 (15%)** | **71 (18%)** |  | **0.08** |  | **71 (18%)** | **85 (22%)** |  | **0.09** |  | **169 (23%)** | **202 (27%)** |  | **0.11** |
| **Tumor grade (WHO 2004)** |  |  |  | **1.00** | **0.02** |  |  |  | **0.70** | **0.04** |  |  |  | **0.25** | **0.07** |
| **Low grade** |  | **8 (2.0%)** | **9 (2.3%)** |  |  |  | **12 (3.1%)** | **15 (3.9%)** |  |  |  | **15 (2.0%)** | **23 (3.1%)** |  |  |
| **High grade** |  | **387 (98%)** | **386 (98%)** |  |  |  | **374 (97%)** | **371 (96%)** |  |  |  | **732 (98%)** | **724 (97%)** |  |  |
| **CIS** |  |  |  | **0.77** | **0.03** |  |  |  | **0.89** | **0.02** |  |  |  | **0.18** | **0.07** |
| **No** |  | **218 (55%)** | **223 (57%)** |  |  |  | **213 (55%)** | **210 (54%)** |  |  |  | **344 (46%)** | **317 (42%)** |  |  |
| **Yes** |  | **177 (45%)** | **172 (44%)** |  |  |  | **173 (45%)** | **176 (46%)** |  |  |  | **403 (54%)** | **430 (58%)** |  |  |
| **Prostate-involving CIS** |  |  |  | **0.80** | **0.04** |  |  |  | **1.00** | **0.02** |  |  |  | **1.00** | **0.00** |
| **No** |  | **386 (98%)** | **388 (98%)** |  |  |  | **379 (98%)** | **380 (98%)** |  |  |  | **732 (98%)** | **732 (98%)** |  |  |
| **Yes** |  | **9 (2.3%)** | **7 (1.8%)** |  |  |  | **7 (1.8%)** | **6 (1.6%)** |  |  |  | **15 (2.0%)** | **15 (2.0%)** |  |  |
| **Divergent differentiation**  **or variants** |  |  |  | **1.00** | **0.00** |  |  |  | **1.00** | **0.02** |  |  |  | **1.00** | **0.00** |
| **No** |  | **385 (98%)** | **385 (98%)** |  |  |  | **376 (97%)** | **377 (98%)** |  |  |  | **724 (97%)** | **724 (97%)** |  |  |
| **Yes** |  | **10 (2.5%)** | **10 (2.5%)** |  |  |  | **10 (2.6%)** | **9 (2.3%)** |  |  |  | **23 (3.1%)** | **23 (3.1%)** |  |  |
| **LVI** |  |  |  | **1.00** | **0.00** |  |  |  | **1.00** | **0.00** |  |  |  | **0.91** | **0.01** |
| **No** |  | **391 (99%)** | **491 (99%)** |  |  |  | **382 (99%)** | **382 (99%)** |  |  |  | **708 (95%)** | **706 (95%)** |  |  |
| **Yes** |  | **4 (1.0%)** | **4 (1.0%)** |  |  |  | **4 (1.0%)** | **4 (1.0%)** |  |  |  | **39 (5.2%)** | **41 (5.5%)** |  |  |
| **Second TUR** |  |  |  | **0.94** | **0.01** |  |  |  | **0.056** | **0.14** |  |  |  | **0.27** | **0.06** |
| **No** |  | **256 (65%)** | **258 (65%)** |  |  |  | **245 (64%)** | **271 (70%)** |  |  |  | **497 (66.5%)** | **518 (69%)** |  |  |
| **Yes** |  | **139 (35%)** | **137 (35%)** |  |  |  | **141 (37%)** | **115 (30%)** |  |  |  | **250 (34%)** | **229 (31%)** |  |  |
| **BCG dose in iBCG** |  |  |  | **0.86** | **0.02** |  |  |  | **0.42** | **0.07** |  |  |  | **0.41** | **0.05** |
| **Full dose** |  | **319 (81%)** | **322 (82%)** |  |  |  | **325 (84%)** | **334 (87%)** |  |  |  | **650 (87%)** | **638 (85%)** |  |  |
| **Reduced dose** |  | **76 (19%)** | **73 (19%)** |  |  |  | **61 (16%)** | **52 (14%)** |  |  |  | **97 (13%)** | **109 (15%)** |  |  |
| **NMIBC, non-muscle invasive bladder cancer; BCG, ; iBCG, induction BCG; mBCG, meintenance BCG; SMD, Standardized mean difference; SD, standard deveation; TURBT, transurethral resection of the bladder tumor; WHO, the World Health Organization; CIS, carcinoma in situ; LVI, lymphovascular invasion; TUR, transurethral resection** | | | | | | | | | | | | | | | |
