# Supplementary material for: Non-maintenance intravesical Bacillus Calmette–Guérin induction therapy with eight doses in patients with high- or highest-risk non-muscle invasive bladder cancer: a retrospective non-randomized comparative study
Source: BMC Cancer. 2021 Mar 11;21:266. doi: 10.1186/s12885-021-07966-7 (PMC7948348; doi:10.1186/s12885-021-07966-7)
Supplement: Supplementary file 5 — Additional file 5: Fig. S2. Landmark analysis of recurrence among patients treated with non-maintenance induction BCG (iBCG) or iBCG plus maintenance BCG (mBCG) at 24 months. Landmark RFS at 6, 12, and 18 months among patients treated with non-maintenance iBCG or iBCG plus mBCG are plotted and compared between matched groups A and B (left panel) and between matched groups B and C (right panel). The P values, hazard ratios (HRs), and 95% confidence intervals (CIs) are shown in the figures. [file 12885_2021_7966_MOESM5_ESM.pptx]

## Slide 1
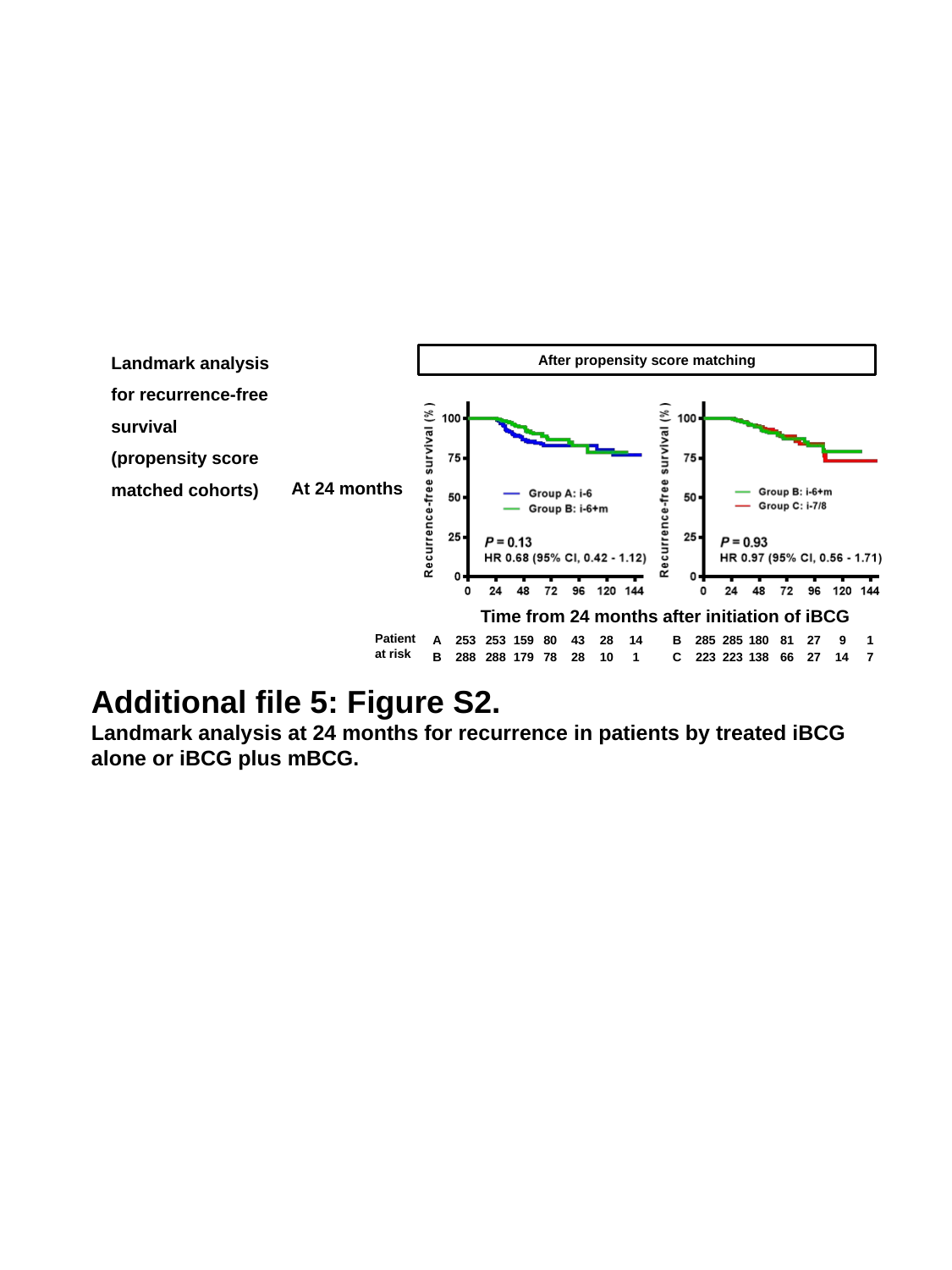

Landmark analysis
for recurrence-free
survival
(propensity score
matched cohorts)
After propensity score matching
At 24 months
Time from 24 months after initiation of iBCG
Patient
at risk
A
253
253
159
80
43
28
14
B
285
285
180
81
27
9
1
B
288
288
179
78
28
10
1
C
223
223
138
66
27
14
7
Additional file 5: Figure S2.
Landmark analysis at 24 months for recurrence in patients by treated iBCG
alone or iBCG plus mBCG.
